# Automatic Speech Recognition and Acoustic Analysis for Dysarthria Assessment in Telerehabilitation: User-Centered Design and Usability Study

**DOI:** 10.2196/85230

**Published:** 2026-07-03

**Authors:** Pierre Vinet, Pierre Dillenbourg, Amelieke Slot, Sharmila Selvanayakam, Sandra Giovanoli, Elisa Du, Julia Cardoso, Meret Branscheidt, Chris Easthope Awai, Christoph Michael Bauer

**Affiliations:** 1 Therapy Science Lab, Health, Lake Lucerne Institute, Rubistrasse 9, Vitznau, 6354, Switzerland, 41 79 5272449; 2 School of Engineering, Section of Microtechniques, École Polytechnique Fédérale de Lausanne, Lausanne, Switzerland; 3 School of Computer amnd Communication Sciences, Computer-Human Interaction Lab for Learning & Instruction, École Polytechnique Fédérale de Lausanne, Lausanne, Switzerland; 4 Center for Neurorehabilitation, Cereneo, Vitznau, Switzerland; 5 Data Analytics & Rehabilitation Technology (DART), Health, Lake Lucerne Institute, Vitznau, Switzerland; 6 Lehre HEST, D-HEST, ETH Zurich, Zürich, Switzerland

**Keywords:** speech and language therapy, user-centered design, telerehabilitation, dysarthria, web application, automatic speech recognition

## Abstract

**Background:**

Dysarthria is a frequent motor speech disorder following a stroke, affecting up to 42% of survivors and resulting in reduced speech intelligibility and diminished quality of life. Clinical assessments, such as the Frenchay Dysarthria Assessment, Second Edition (FDA-2), rely heavily on the subjective judgment of speech-language pathologists (SLPs), which limits comparability and scalability. Telepractice solutions have the potential to extend access to care, but validated digital tools that combine automatic analysis with clinically usable interfaces remain scarce.

**Objective:**

This study aimed to develop and evaluate a web-based application that integrates automatic speech recognition (ASR) and acoustic analysis into a user-centered dashboard for SLPs. Specifically, we investigated: (1) whether ASR can provide intelligibility scores comparable to those of human listeners; (2) the usability of the system in 2 iterative cycles with SLPs; and (3) the feasibility of presenting clinically relevant acoustic features to support telerehabilitation.

**Methods:**

A user-centered design process was followed, involving contextual inquiry, requirements gathering, prototype development, and iterative testing with SLPs. The analytical core of the prototype included an ASR module (Whisper Large-v3) to compute intelligibility scores, combining word error rate–based accuracy with sentence-level and word-level alignment. Phoneme-level error highlighting was implemented to identify frequent substitution or deletion patterns. In parallel, an acoustic module extracted clinically relevant measures, including fundamental frequency (mean and range), intensity (mean and variability), and vowel formants (F1–F2 space), supplemented by sustained phonation duration. A pilot validation compared ASR-based intelligibility scores with transcriptions from 8 lay listeners for 3 patients with dysarthria performing the Frenchay Dysarthria Assessment–2 word and sentence tasks. Usability was evaluated in 2 cycles with 8 and 4 SLPs, respectively, using the System Usability Scale and structured questionnaires.

**Results:**

In the pilot validation, ASR performance was comparable to, and in some cases better than, untrained human listeners for individuals with mild and moderate dysarthria, though performance declined with severe cases. Both usability cycles yielded excellent System Usability Scale scores (cycle 1: mean 88.4, SD 4.6; cycle 2: mean 91.7, SD 4.1). Core workflow elements, including navigation, session upload, and intelligibility score presentation, were consistently rated highly. Feedback evolved from bug reports and requests for clearer terminology in cycle 1 to suggestions for advanced analytic features in cycle 2, such as additional voice-quality indices and integrated note-taking.

**Conclusions:**

The prototype demonstrates that automatic intelligibility scoring and acoustic analysis can be integrated into a clinically usable, web-based dashboard. While current limitations include reliance on English-only phoneme analysis, limited advanced acoustic features, and lack of regulatory compliance, the application achieved excellent usability and shows promise for scalable telerehabilitation. Future work should expand multilingual support, incorporate additional acoustic measures, and validate the tool in larger clinical cohorts.

## Introduction

Dysarthria is a neuromotor speech disorder resulting from neurological damage and is present across many neurological diseases, including stroke, cerebral palsy, and amyotrophic lateral sclerosis [1,2]. It affects the speed, strength, accuracy, range, tone, or duration of the movements required for speech control and commonly reduces speech intelligibility [1], substantially impacting participation, psychosocial well-being, and quality of life [3,4]. Clinical assessment tools, such as the Frenchay Dysarthria Assessment, Second Edition (FDA-2), one of the most widely used assessments across different clinical systems [5], are widely used but rely heavily on subjective judgment, leading to interrater and intrarater variability in some items [6-8]. This creates variability and limits comparability across raters and time.

Speech-language pathologists (SLPs) play a vital role in diagnosing and treating dysarthria [9], yet their reach is constrained by time, geography, and patient accessibility. Telerehabilitation, which involves delivering rehabilitation services remotely, has emerged as a scalable, clinically endorsed solution to bridge this gap, with professional guidance and reimbursement pathways increasingly being established [9-12]. By enabling remote assessment and treatment, telerehabilitation can extend therapeutic support to underserved regions and improve continuity of care for individuals who experienced stroke.

Integrating acoustic analysis based on automatic speech recognition (ASR) into telerehabilitation platforms opens the possibility of continuous, noninvasive speech monitoring in naturalistic settings and timely feedback to patients. Advancements in signal processing and large self-supervised “audio or language” models now enable robust extraction of acoustic features (eg, fundamental frequency, intensity range, and speaking rate) and interpretation of speech. Such measures serve as quantifiable indicators of speech quality and progression, specifically the severity of dysarthria, providing data-driven support to SLPs and researchers during evaluation [13-15]. Modern representation-learning approaches [16] further strengthen automated analyses and downstream assessment pipelines [17-19]. However, the integration of these advances into clinical practice has not kept pace, creating a mismatch between technological potential and clinical uptake that motivates this study. Despite these advances, adoption in routine care remains limited. Many available tools require installing desktop software and substantial technical training, have limited usability, fit poorly within clinical workflows, and lack standardized, shareable digital outcome measures. Furthermore, patient privacy needs and data protection concerns must be addressed. As a result, human perceptual judgment continues to serve as the de facto reference standard [17,20,21].

This study addresses this gap by developing a web-based tool that integrates ASR and acoustic analysis. While initially designed with stroke-related dysarthria in mind [22], the underlying analytical framework is intended to be generalizable across dysarthria etiologies. Specifically, we examined: (1) whether ASR can achieve comparable performance to human listeners in intelligibility assessment; (2) the usability of the tool for SLPs in a formative evaluation; and (3) implications for telerehabilitation and long-term monitoring.

## Methods

### Study Design

A user-centered design approach guided the project from early requirements gathering to final prototype validation [23,24]. Initial requirements were collected before the frontend design to match the SLPs’ requirements for a web-based digitized speech and language assessment [22]. This study consisted of a three-step approach: (1) needs and context analysis including specifying the context of use and gathering user requirements; (2) preliminary testing; and (3) end-user testing and refinement. The 2 empirical components—prototype development and preliminary testing, and end-user testing and refinement—are reported in the “Results section.” Two iterative usability testing cycles were conducted to identify and address initial usability issues and to evaluate the effectiveness of implemented improvements in a refined prototype.

### Ethical Considerations

Patient data were obtained from patients with stroke and the publicly available TORGO database, a database that includes individuals with cerebral palsy and amyotrophic lateral sclerosis, which permits academic use [25]. All procedures adhered to ethical guidelines and regulations. Approvals were obtained from the Ethics Committee of Northwestern and Central Switzerland (Req-2024‐00103 for usability testing, R-2025‐00538 for voice recordings). Written informed consent was obtained from all participants. The participants did not receive compensation.

### User-Centered Design

#### Needs and Context Analysis

##### Needs Assessment and Context Analysis

The iSpeak system was initially developed for SLPs conducting assessments of dysarthria in individuals who experienced stroke [22]. To capture dysarthria across neurological conditions more broadly, this study used the TORGO database [25]. Nine SLPs were recruited, and participant demographics are summarized in Table 1. A purposive sampling method was used, based on the assumption that each participant would offer unique insights [26]. Because the participants’ roles were not interchangeable, the sample size was guided by data saturation rather than statistical power analysis [26]. The SLPs contributed through preliminary meetings, focus groups, prototype testing, and feedback.

The following 2 methods informed contextual understanding:

- Work shadowing sessions (n=6, 30‐45 min each), including observation of live telerehabilitation sessions and mock sessions where 1 researcher acted as the patient, revealed workflow challenges and opportunities for automation. Field notes from the work shadowing sessions were summarized descriptively and reviewed to identify recurring workflow steps, contextual constraints, and opportunities for automation using Microsoft Excel.
- Two focus group interviews with a total of 6 SLPs, who practiced telerehabilitation with a mean experience of 4.6 years, were conducted to explore assessment practices, workflow bottlenecks, and requirements for automated tools. Focus group responses were summarized descriptively by the research team and reviewed to identify recurring needs, workflow barriers, and potential requirements for the prototype; no dedicated qualitative analysis software was used for this step. During the focus groups, the SLPs emphasized the need for asynchronous use. Typical practice involved conducting a live therapy session via videoconferencing, followed by posthoc review and analysis using the application.

**Table 1.** Overview of participants in study phases.

Phase	Participants	Region	sex (female), n	Work experience (y), mean (SD; range)
Needs and context analysis				
Work shadowing	3 patients and 3 mock sessions	Europe (4)–Arabic Peninsula (1)	3	—^a^
Focus group interviews	6 SLPs^b^	Europe (4)–Arabic Peninsula (1)	5	4.6 (2.1; 3-6)
User requirements	6 SLPs	Europe (4)–Arabic Peninsula (1)	5	4.6 (2.1; 3-6)
Preliminary testing				
Pilot validation	8 lay users	Europe (8)	5	—
End-user testing and refinement				
Usability study cycle 1	8 SLPs	Europe (7)–United States (1)	8	7.56 (3.2; 3-26)
Usability study cycle 2	5 SLPs	Europe (4)–Arabic Peninsula (1)	5	4.3 (2.0; 3–6)

a Not applicable

b SLP: speech and language pathologist.

##### User Requirements

Requirements were identified from observations and focus groups, summarized by the research team, and classified into functional and nonfunctional categories (Tables S6-S8 in Multimedia Appendix 1). The requirements were then prioritized according to feasibility and perceived importance for SLPs using Microsoft Excel; functional requirements described expected features (eg, review of recordings, intelligibility scoring, and progress tracking), while nonfunctional requirements addressed qualities such as speed, reliability, and security. Prioritization considered both feasibility and user importance. These requirements informed prototype design and evaluation (Tables S6-S8 in Multimedia Appendix 1).

##### Design and Prototype Development

**Figure 1.**
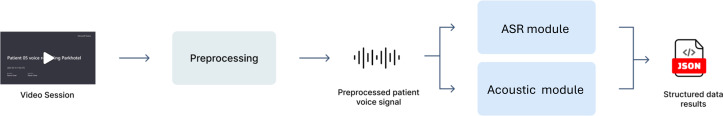
Overview of the backend modules for the analysis end point. ASR: automatic speech recognition.

The prototype design was created in Figma using Shadcn component libraries to ensure consistency and rapid iteration. Designs were validated with SLPs before implementation. The final prototype comprised a backend (Python, Python Software Foundation; FastAPI, Sebastián Ramírez [known online as @tiangolo]; PostgreSQL, PostgreSQL Global Development Group) hosted on a Swiss VPS, and a frontend (Next.js, Vercel Inc; Tailwind CSS, Tailwind Labs Inc) deployed via Vercel, Vercel Inc. These implementation details are provided in Multimedia Appendix 1. Figure 1 illustrates the prototype's backend modules for the analysis end point. The prototype's style guide is illustrated in Figure S6 in Multimedia Appendix 1.

### Evaluation of the Prototype

The prototype was evaluated in 2 stages: prototype development and preliminary testing with lay users to detect usability issues and bugs, as well as the technical validity of ASR-based intelligibility scoring; and end-user testing and refinement with SLPs across 2 iterative cycles, during which SLPs evaluated usability, workflow integration, and clinical relevance.

### Preliminary Testing

Local community members familiar with laptops explored the prototype freely, uploading recordings and navigating the dashboard. A mock session ensured comparability. Minor issues (eg, broken links and unclear navigation) were corrected before expert testing.

#### Participants

Eight lay listeners were recruited from the faculty staff. None had training in speech-language pathology, but all possessed advanced English proficiency. They were selected as proxies for naïve intelligibility judgments, which are commonly used in dysarthria research to approximate real-world listener understanding rather than relying on expert clinical ratings [27]. Three patients with stroke with varying dysarthria severity were recruited from a neurorehabilitation hospital and provided speech material. Each had completed the FDA-2 reading tasks (10 words and 10 sentences) [5].

#### Materials and Procedure

Recordings were processed using OpenAI Whisper (Large-v3), configured for English with a temperature of 0.0 [28]. Participants listened via a laptop and transcribed the speech without access to reference texts. Both lay transcriptions and ASR output were normalized using the same procedures and compared against reference texts. Performance was quantified by word error rate (WER), with lower WER indicating higher intelligibility. The results were visualized using boxplots.

### End-User Testing and Refinement

### Usability Testing and Refinement

#### Overview

Two structured cycles followed a standardized protocol. Each participant received access credentials, a user manual with screenshots, and sample patient data (from TORGO). Usability was assessed through a questionnaire that included open-ended questions, Likert scales, and the System Usability Scale (SUS) [29]. Anonymous web analytics complemented self-reports by capturing interaction patterns and navigation behavior. Likert-scale questionnaire items and SUS scores were analyzed descriptively by calculating item-level and overall mean scores. Open-ended questionnaire responses were summarized and categorized according to their content using Microsoft Excel. Anonymous web analytics were inspected descriptively to identify navigation patterns, task completion issues, and dead clicks. After each cycle, open-ended feedback and observed usability issues were categorized into bugs, improvements, and feature requests, which were used to guide the next refinement step. Critical issues were fixed before the next cycle. The analytical pipeline was designed to be condition-agnostic, operating on speech signal characteristics rather than disease-specific features, thereby supporting potential generalization across different dysarthria etiologies.

Results of the 2 cycles, including SUS scores and qualitative feedback, are presented in the “Results” section.

#### Participants

Eight SLPs who practiced telerehabilitation were recruited through an established professional network to participate in 2 usability cycles, with a mean of 7.57 years of clinical practice. The SLPs used the application at their workplaces or homes. The SLPs received audio recordings and reference transcripts from the TORGO database [25] to ensure compliance with health care patient data regulations while providing realistic clinical testing scenarios.

### The Prototype

#### Preprocessing

Recordings were preprocessed by extracting audio, applying a band-pass filter (80 Hz-8 kHz), normalizing the signal amplitude, and resampling to 16 kHz mono. These steps ensured consistency across sessions and compliance with model requirements [28].

#### Transcription and Normalization

Whisper Large-v3 produced transcriptions, which, together with reference texts, were normalized (lowercased, punctuation removed, numbers expanded, and contractions expanded) to ensure scoring reflected content rather than formatting [30,31].

#### Error Metrics

WER and character error rate were calculated using the standard edit-distance (Levenshtein) algorithm. WER is defined as the sum of substitutions, deletions, and insertions divided by the total number of reference words. Character error rate follows the same logic at the character level. Detailed equations are provided in Multimedia Appendix 1 [31].

#### Alignment

To compute sentence-level scores, a global alignment between concatenated reference and transcription strings was performed. Insertions, deletions, and substitutions were marked, and then boundaries were adjusted back to individual sentences. This allowed for per-sentence WER and alignment visualizations highlighting correctly and incorrectly recognized words.

#### Intelligibility Score

Intelligibility refers to how much of a speaker’s intended message is understood by a listener and is commonly operationalized in dysarthria research through listener transcription accuracy at the word or sentence level [13,27,32,33]. In FDA-2 scoring, each item is judged as correct or incorrect. We implemented two scoring modes:

- Binary scoring (FDA-2 standard) [5]: words and sentences are scored as correct or incorrect.
- Word-level scoring: sentence scores are calculated from the average word-level accuracy.

The final intelligibility score (IS) was defined as follows:

$IS=1-WER_{\text{avg}}$(1)

Where WER_avg_ is the average WER across tasks. Global scores combine word and sentence results as follows:

$IS_{global}=\frac{N_{w}*IS_{w}+N_{s}*IS_{s}}{N_{w}+N_{s}}$(2)

where *IS_W_* and *IS_S_* are word and sentence intelligibility scores, and *N_w_* and *N_s_* are their respective counts.

#### Phoneme-Level Analysis

Phonemes are the smallest contrastive speech sound units that can distinguish meaning within a language; phoneme-level analysis, therefore, focuses on the sound structure of words rather than on the listener’s global understanding of the message [34]. Reference words were converted to phoneme sequences using CMUdict and mapped to the International Phonetic Alphabet. Errors were classified as substitutions or deletions, aggregated, and ranked by frequency and word position. This analysis provided SLPs with clinically relevant insights for tailoring therapy.

#### Acoustic Feature Extraction

Acoustic features were extracted to provide objective, interpretable measures of speech. Based on clinical input, 3 families were prioritized as follows [35-37]:

- Fundamental frequency (F0): mean, range, and variability.
- Intensity: mean and SD.
- Formants (F1, F2): vowel-space measures that indicate articulatory precision.

Maximum phonation time was derived from sustained /a/ tasks. These features capture prosodic control, respiratory support, and articulation. Sex-related differences in vocal tract anatomy, which influence formant frequencies (F1 and F2), were not controlled for in this study. Extraction was performed using Parselmouth, a Python interface to Praat, integrated into the backend pipeline.

#### Statistical Methods Used for Analyzing Acoustic Features

The extracted acoustic features were computed using Parselmouth and summarized descriptively to support clinical interpretation. No inferential statistical analyses were performed on these features, as the study focused on the feasibility and usability of the analytical pipeline rather than a hypothesis-driven evaluation of acoustic measures.

#### Dashboard and Visualization

The web application dashboard was designed to present results clearly to SLPs. Key components included (1) circular progress indicators showing intelligibility percentages; (2) bar charts of phoneme error frequencies, color-coded by word position; and (3) side-by-side alignment displays highlighting correctly recognized (green) versus incorrect (red) words.

**Figure 2.**
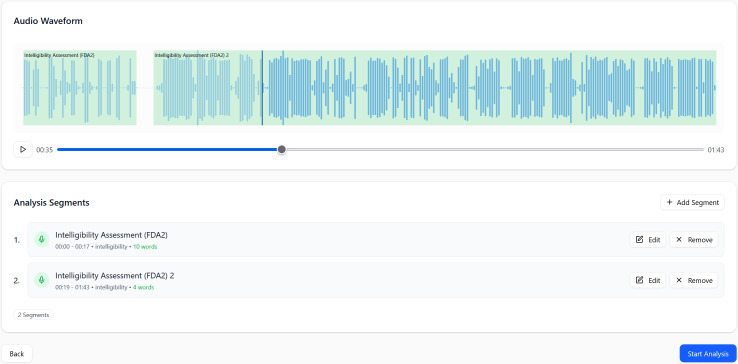
Analysis and segment selection (of 1 TORGO database patient). FDA2: Frenchay Dysarthria Assessment, Second Edition.

Figures2^4^ illustrate the interface components and analytics dashboards.

**Figure 3.**
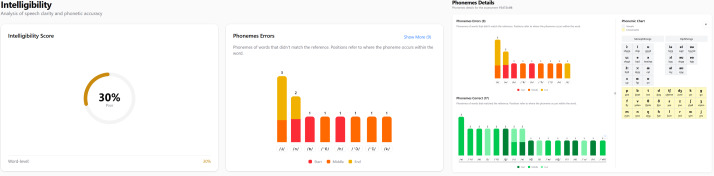
Intelligibility score and phonemes analysis (left) detailed phonemes analysis page (right) of a patient from the TORGO database.

**Figure 4.**
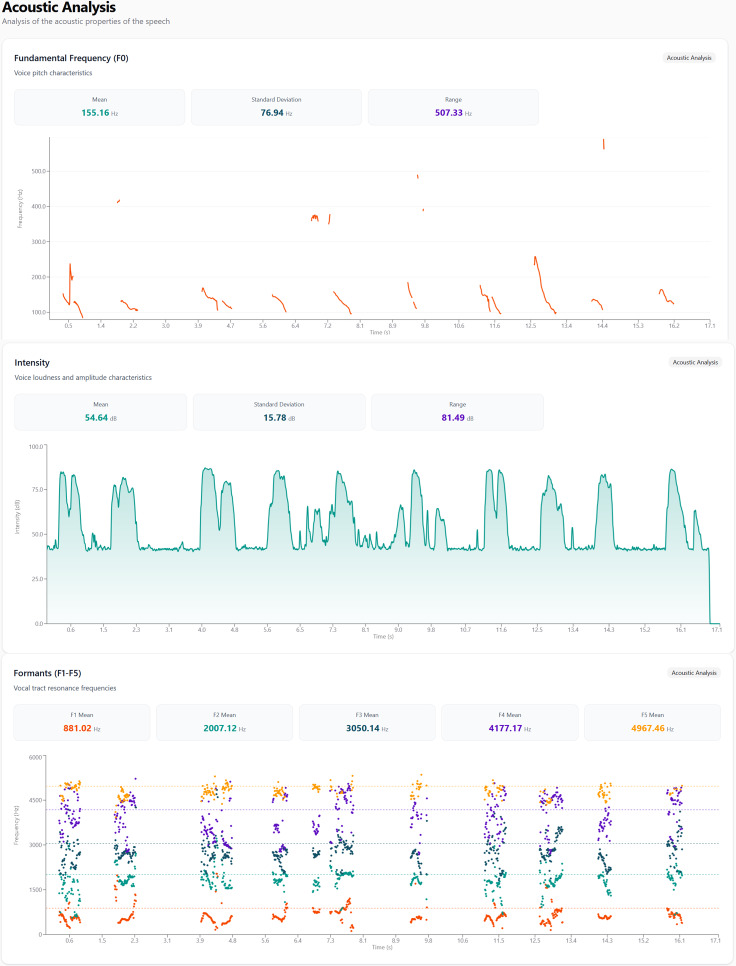
Acoustic analysis: acoustic base frequency (top), acoustic intensity (middle), acoustic formants (bottom) of a patient from the TORGO database. F0: acoustic base frequency, F1-F5: acoustic formants.

## Results

Table 1 provides an overview of the study phases and participant demographics. All SLPs who took part in the study practice telerehabilitation.

### Preliminary Testing

Eight lay users (Table 1) transcribed audio recordings from 3 patients with dysarthria to validate the accuracy of the automatic intelligibility assessment. The recordings included the FDA-2 words and sentences tasks. Performance was quantified using WER, with lower values indicating greater accuracy.

**Figure 5.**
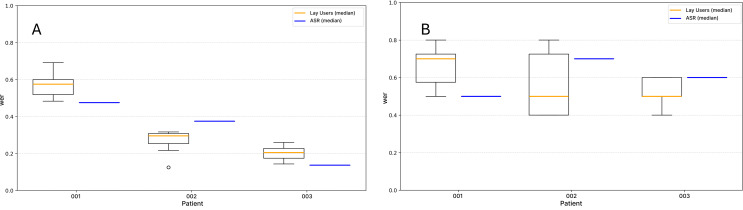
Word error rate (WER) comparison between lay user transcriptors and ASR transcriptions for the word (A) and sentences (B) tasks; ASR: automatic speech recognition system.

The ASR system (Whisper Large-v3) performed comparably to, and in some cases better than, untrained listeners. For mild dysarthria, ASR achieved lower median WERs than most participants. For moderate and severe dysarthria, ASR errors increased but remained within the variability range of human listeners. Boxplots of WER distributions are shown in Figure 5.

These findings confirm that ASR can approximate human perceptual judgments of intelligibility and support its integration into the prototype.

### End-User Testing and Refinement-Cycle 1

Eight SLPs participated in the first usability cycle (mean clinical experience 7.56, SD 3.2 y; Table 1). Most accessed the application at the workplace, while 2 participated from home, using a mix of Chrome, Firefox, and Edge.

The mean SUS score was 88.4 (SD 4.6), which is considered excellent [38,39]. Average ratings across survey items were 4.26/5 (Table 2). Core workflow elements—including navigation, uploading sessions, segment selection, and processing time—scored between 4.50 and 4.63. Visual design received the highest rating (mean 4.88, SD 0.35). Understanding the intelligibility outputs scored slightly lower: the side-by-side transcription view was rated 4.50, and the average intelligibility score clarity was 4.13 (SD 0.13). The lowest ratings were for the perceived accuracy of the automatic intelligibility score (mean 3.13, SD 1.25) and phoneme-level error highlighting (mean 3.88, SD 1.25). Qualitative feedback (Table 3; Table S9 in Multimedia Appendix 1) clustered into 3 categories:

- Fixes or bugs: reliability and alignment issues (failed uploads, shifting regions, and missed words).
- Usability improvements: clearer wording in the user interface, simultaneous listening and text entry, and time markers.
- Feature requests: phonetic equivalence in intelligibility scoring, and pause analysis.

These results confirmed high usability while identifying areas for refinement before the second cycle.

**Table 2.** End-user testing and refinement—cycle 1.

Survey question	SLP^a^ score (out of 5), mean (SD)
The web application met my expectations.	3.88 (1.13)
The navigation within the app was easy and logical.	4.50 (0.76)
The design of the app (colors, fonts, layout) was appealing and professional.	4.88 (0.35)
The session upload process was straightforward	4.63 (0.74)
Selecting audio segments was intuitive	4.63 (0.52)
The time required for upload and selection was acceptable	4.50 (0.53)
How easy was it to select audio segments and upload your video or audio session?	4.50 (1.41)
The automatic intelligibility score seemed accurate	3.13 (1.25)
The intelligibility score was easy to understand	4.13 (0.99)
The side-by-side view of patients` transcription versus reference was clear	4.50 (0.76)
The phoneme-level error highlighting was intuitive	3.88 (1.25)
How easy was the intelligibility score to understand?	4.00 (2.51)
Overall mean across questions	4.26 (0.58)

a SLP: speech and language pathologist.

**Table 3.** End-user testing and refinement—cycle 1.

ID^a^	Representative SLP^b^ feedback	Category
I01-002	First files did not upload.	Fix or bug
I01-013	The selected region shifted right when I extended the clip.	Fix or bug
I01-004	The term “misspelled” is confusing because it suggests the SLP made the error.	Improvement
I01-005	I want to listen to the segment while I enter the reference words.	Improvement
I01-007	Show minutes/seconds under the clip to guide word and sentence selection.	Improvement
I01-019	Count phonetic equivalents as correct in the intelligibility calculation.	Feature
I01-018	Analyze the duration of pauses between words to reflect prosody issues.	Feature

a ID: feedback identifier.

b SLP: speech and language pathologist.

### End-User Testing and Refinement-Cycle 2

Five SLPs participated in the second usability cycle (mean clinical experience 4.3, SD 2.0 y; Table 1). The testing environments and browsers were the same as those used in cycle 1.

The mean SUS score increased to 91.7 (SD 4.1), which is again considered excellent [38]. Survey results (Table 4) showed consistently high ratings. Navigation and the new acoustic analysis page both received a score of 4.75, indicating that participants found the workflow intuitive and the additional analysis features useful. Frequency and intensity graphs were rated positively (4.33 each), while the formants graph was rated lower (mean 3.66, SD 0.65), suggesting limited clarity or perceived clinical utility. Overall expectations were rated at a mean of 4.20 (SD 0.42).

**Table 4.** End-user testing and refinement–cycle 2.

Survey question	SLP^a^ score (out of 5), mean (SD)
I found the fundamental frequency graph useful.	4.33 (0.57)
I found the intensity graph useful.	4.33 (0.89)
I found the formants graph useful.	3.66 (0.65)
How easy was the acoustic analysis page to understand?	4.75 (0.41)
The web application met my expectations	4.20 (0.42)
The navigation within the app was easy and logical.	4.75 (0.78)
Overall mean across questions	4.34 (0.40)

a SLP: speech and language pathologist.

Representative feedback (Table 5) emphasized advanced feature requests. Suggestions included the option to add notes to assessment results, integration of advanced voice-quality indices (eg, Cepstral Peak Prominence [CPPS] and Acoustic Voice Quality Index [AVQI]), and audio playback directly from result pages. These comments indicate readiness for clinical refinement, with priorities shifting toward advanced analysis rather than fundamental usability.

**Table 5.** End-user testing and refinement–cycle 2.

ID^a^	Representative SLP^b^ feedback	Category
I02-002	Ability to add notes to an assessment result. It should be possible to comment on patient behavior (eg, “struggled with breath here”).	Feature
I01-013	Inclusion of voice-quality indices (eg, CPPS^c^ and AVQI^d^) was suggested to provide additional clinical insight.	Feature
I01-004	Playback of the analyzed audio directly from the Results page was requested to allow comparison between perceived audio and computed metrics.	Feature
I01-005	Ability to listen to the uploaded audio on the Results page was requested, especially to review the alignment between the reference text and the transcription	Feature

a ID: feedback identifier.

b SLP: speech and language pathologist.

c CPPS: Cepstral Peak Prominence.

d AVQI: Acoustic Voice Quality Index.

### Summary of Results

Across all 3 substudies, the iSpeak prototype demonstrated promising performance and usability. The pilot validation confirmed that ASR achieved accuracy comparable to or greater than that of untrained human listeners across varying severities of dysarthria. Both usability cycles with SLPs yielded excellent SUS scores (>88), with cycle 2 reaching a mean of 91.7 (SD 4.1) after refinement. Core workflows were consistently rated highly, and feedback evolved from identifying technical issues in cycle 1 to requesting advanced analytical features in cycle 2. Together, these results indicate that the system is both usable and potentially clinically relevant, with future development focused on enhanced analytic capabilities and broader validation.

## Discussion

### Principal Findings

This study evaluated a web-based application designed to support SLPs in assessing dysarthric speech in patients with poststroke. Developed using a user-centered design framework in accordance with the International Organization for Standardization (ISO) 9241‐210, the prototype integrates ASR and acoustic analysis into a clinical dashboard. A pilot validation compared ASR with lay listeners, followed by 2 iterative usability cycles with SLPs. The findings demonstrate that ASR can approximate human performance in intelligibility assessment and that the application was consistently rated as highly usable, with SUS scores above 85 in both cycles. Feedback confirmed the value of automatic intelligibility scoring and acoustic analysis while identifying priorities for further development.

### Preliminary Testing

The pilot validation provided initial evidence of how ASR compares with human listeners on dysarthric speech. Across tasks, word lists produced higher WERs than sentence lists, reflecting the contextual advantage of sentences [27]. Importantly, lay listeners were not native English speakers, which may have introduced bias, as subtle dysarthric articulations could be more difficult to interpret for nonnative listeners [40]. This may have led to an underestimation of intelligibility relative to trained or native listeners [41].

Patient-level differences were evident. For “Patient 001,” ASR outperformed most lay listeners across tasks, suggesting that the system could handle this speech relatively well. For “Patient 003,” ASR performance was competitive, especially in the sentences task. “Patient 002” posed the greatest challenge: lay listeners showed high variability in WERs for the word task, and ASR performed worse than all listeners in the sentences task. These findings highlight how severity and individual variability in dysarthria strongly affect ASR performance [27,40,41].

Although Whisper achieves state-of-the-art performance on typical speech, its accuracy declines with dysarthric input. Stroke-related dysarthria alters pronunciation, speech rate, and voice quality in diverse ways. Because such data are underrepresented in training corpora, ASR robustness remains limited [32,41,42]. Nonetheless, pilot results indicate that Whisper can sometimes match or surpass untrained human listeners, suggesting promise as a baseline for automatic intelligibility scoring. Similar findings are reported in recent validation efforts of automatic intelligibility measures for motor speech disorders [43]. Future validation with larger patient cohorts and SLP raters is needed to confirm these observations.

### End-User Testing and Refinement–Cycle 1

The first usability cycle demonstrated that the prototype was already perceived as highly usable. The mean SUS score of 88.4 (SD 4.6) falls in the “excellent” range, confirming that the design was well-adapted to SLPs’ needs [44]. Importantly, the application was tested both in clinical and home environments, as well as across different browsers, indicating technical robustness in varied contexts of use.

Survey ratings averaged 4.26/5. Core workflow elements—navigation, uploading, segment selection, and processing time—scored above 4.5, and visual design was rated highest (mean 4.88, SD 0.35). These results align with reviews of existing speech therapy apps, which similarly show that visual design and general usability are often rated highly while output clarity (such as the accuracy of automated scores or error highlighting) tends to receive more mixed feedback [38,45].

Three items scored lower: perceived accuracy of the intelligibility score (mean 3.13, SD 1.25), phoneme-level error highlighting (mean 3.88, SD 1.25), and meeting expectations (mean 3.88, SD 1.13). These reflected a technical alignment bug in this prototype version, which caused intelligibility scores and error highlights to miss clearly spoken words. This limitation directly influenced perceptions of accuracy, a pattern also reported in empirical studies of online SLP tools, where users frequently note misalignments or ambiguous output explanations [39].

Open feedback aligned with these quantitative findings. Fixes focused on reliability (eg, failed uploads and region shifting). Usability improvements included clearer terminology, simultaneous listening and transcription, and visible time markers. Feature requests suggested phonetic equivalence in scoring and pause analysis. Similar issues of clinician-perceived usability and workflow integration have been noted in broader reviews of automated speech therapy tools [45,46]. Fourteen priority fixes and improvements were implemented for cycle 2, balancing feasibility with the need to proceed rapidly to the next evaluation.

### End-User Testing and Refinement–Cycle 2

The second cycle reinforced and extended these findings. The mean SUS score increased to 91.7 (SD 4.1), again in the “excellent” range, suggesting that refinements made after cycle 1 successfully improved usability, as SLP tools evolve from basic usability to richer features [44,45]. As before, testing across home and clinical settings and multiple browsers confirmed robustness. This pattern mirrors findings in reviews of eHealth speech-language therapy applications, where iterative improvements based on clinician feedback tend to yield measurable increases in satisfaction and functionality [38,45].

Survey ratings averaged 4.34/5. Navigation and the new acoustic analysis page received the highest scores (mean 4.75, SD 0.78). Frequency and intensity graphs were positively rated (4.33 each), confirming clinical relevance. The formants graph was rated lower (mean 3.66, SD 0.65), indicating that its value was less evident in practice. The “meeting expectations” item improved to a mean of 4.20 (SD 0.42). These improvements in visual clarity and interactive analytics reflect the user experience findings from telepractice tools, where dashboards and visualization features are increasingly valued as maturity grows [47].

Qualitative feedback shifted from identifying bugs to requesting advanced features. Suggestions included adding notes to assessment results, incorporating voice-quality indices such as CPPS and AVQI, and enabling audio playback directly from results pages. This shift is consistent with empirical studies of speech therapy platforms, where, once baseline usability is achieved, user requests focus more on analytic depth and interactivity [20,48]. Broader surveys of online and AI-enabled speech therapy systems echo this pattern, with early iterations focusing on usability and later development emphasizing advanced functionality [45,46].

### The Prototype

The final prototype can be considered a simple, efficient, and potentially clinically relevant dashboard for assessing speech clarity and phonetic accuracy. Usage analytics confirmed the intuitive design: task times decreased after initial use, and only 3 dead clicks were recorded. The modular architecture enables extension to multilingual contexts, although current phoneme segmentation is restricted to English. While Whisper is multilingual, the phoneme module relies on English-only resources, limiting its applicability across languages.

Occasional ASR hallucinations were observed in cases of severe dysarthria or poor microphone quality. These issues were mitigated through design decisions (eg, suppressing insertions in alignment), ensuring that phoneme analysis was not distorted. The current implementation also assumes one speaker per session; while speaker diarization could be added to handle overlapping speech, it introduces additional complexity.

### Future Work

Several directions for development emerged. First, multilingual phoneme support is required to reflect the diversity of clinical populations. Second, advanced acoustic indices (eg, jitter, shimmer, CPPS, and AVQI) should be integrated to capture additional aspects of dysarthria severity [35-37]. Third, diarization would allow for the analysis of multispeaker sessions, accounting for overlaps or caregiver contributions. Finally, larger-scale validation with patients and practicing SLPs is essential to confirm accuracy and usability across broader contexts. An important next step is to systematically evaluate the generalizability of the system across different neurological populations, as initial testing across mixed etiologies suggests feasibility but does not yet establish condition-specific validity. Future work should also consider implementation within the broader framework of telerehabilitation, where evidence is growing for the effectiveness of remote interventions in speech-language therapy [49].

### Limitations

At present, the system is not compliant with data protection regulations such as the Health Insurance Portability and Accountability Act (HIPAA) and the Health Information Technology for Economic and Clinical Health Act (HITECH), which poses a barrier to clinical deployment over the internet. Use within closed clinical networks is feasible, but full compliance will be essential for wider adoption. A limitation of this study is the small sample size in the usability testing cycles, which may limit the generalizability of the usability findings and warrants further testing in larger cohorts.

### Conclusion

This study demonstrates the feasibility of integrating ASR and acoustic analysis into a web-based application to support SLPs in assessing dysarthric speech. A pilot validation confirmed that ASR performance was comparable to that of untrained human listeners, while 2 usability cycles with SLPs yielded consistently excellent SUS scores, indicating that the system was perceived as highly usable and clinically relevant. Iterative refinements improved navigation and workflow, and feedback evolved from bug reports to requests for advanced analytic features, underscoring both the robustness of the core design and the demand for deeper functionality. Although current limitations include reliance on English-only phoneme segmentation, the prototype establishes a solid foundation for scalable digital assessment. Future work should extend validation to larger and more diverse patient groups, expand multilingual support, and integrate additional advanced outcome measures to further enhance clinical adoption and impact.

## Supplementary material

10.2196/85230 Multimedia Appendix 1 Needs, requirements, feedback, and style guide.

## Abbreviations

ASR: automatic speech recognition
AVQI: Acoustic Voice Quality Index
CPPS: Cepstral Peak Prominence
FDA-2: Frenchay Dysarthria Assessment, Second Edition
HIPAA: Health Insurance Portability and Accountability Act
HITECH: Health Information Technology for Economic and Clinical Health Act
ISO: International Organization for Standardization
SLP: speech and language pathologist
SUS: System Usability Scale
WER: word error rate

## References

[1] Jayaraman DK, Das JM (2023). StatPearls.

[2] Dysarthria in adults. American Speech-Language-Hearing Association.

[3] Vogel AP, Graf L, Weiß M, Chan CSJ, Hepworth G, Synofzik M (2026). Development and validation of the dysarthria impact scale: a patient-reported outcome for motor speech disorders. J Neurol.

[4] Atkinson-Clement C, Letanneux A, Baille G (2019). Psychosocial impact of dysarthria: the patient-reported outcome as part of the clinical management. Neurodegener Dis.

[5] Enderby P (1980). Frenchay Dysarthria Assessment. Int J Lang Commun Disord.

[6] Riolo V, Pizzorni N, Guanziroli E (2022). Cross-cultural adaptation into Italian and validation of the Frenchay Dysarthria Assessment - 2. Eur J Phys Rehabil Med.

[7] Cardoso R, Guimarães I, Santos H (2017). Frenchay Dysarthria Assessment (FDA-2) in Parkinson’s disease: cross-cultural adaptation and psychometric properties of the European Portuguese version. J Neurol.

[8] Icht M, Bergerzon-Bitton O, Ben-David BM (2022). Validation and cross-linguistic adaptation of the Frenchay Dysarthria Assessment (FDA-2) speech intelligibility tests: hebrew version. Int J Lang Commun Disord.

[9] Telepractice. American Speech-Language-Hearing Association.

[10] Telehealth guidance. Royal College of Speech and Language Therapists.

[11] What is speech? What is language?. American Speech-Language-Hearing Association.

[12] Scott AM, Clark J, Cardona M (2025). Telehealth versus face-to-face delivery of speech language pathology services: a systematic review and meta-analysis. J Telemed Telecare.

[13] Yorkston KM, Strand EA, Kennedy MRT (1996). Comprehensibility of dysarthric speech: implications for assessment and treatment planning. Am J Speech Lang Pathol.

[14] Kent RD, Kim YJ (2003). Toward an acoustic typology of motor speech disorders. Clin Linguist Phon.

[15] Kim Y, Kim M, Kim J, Song TJ (2024). Efficacy and feasibility of a digital speech therapy for post-stroke dysarthria: protocol for a randomized controlled trial. Front Neurol.

[16] Baevski A, Zhou H, Mohamed A, Auli M (2020). Wav2vec 2.0: a framework for self-supervised learning of speech representations. NIPS’20: Proceedings of the 34th International Conference on Neural Information Processing Systems.

[17] Tucker JK (2012). Perspectives of speech-language pathologists on the use of telepractice in schools: the qualitative view. Int J Telerehabil.

[18] Hsu WN, Bolte B, Tsai YHH, Lakhotia K, Salakhutdinov R, Mohamed A (2021). HuBERT: self-supervised speech representation learning by masked prediction of hidden units. IEEE/ACM Trans Audio Speech Lang Process.

[19] Chen S, Wang C, Chen Z (2022). WavLM: large-scale self-supervised pre-training for full stack speech processing. IEEE J Sel Top Signal Process.

[20] Molini-Avejonas DR, Rondon-Melo S, Amato CA, Samelli AG (2015). A systematic review of the use of telehealth in speech, language and hearing sciences. J Telemed Telecare.

[21] Mitchell J, Shirota C, Clanchy K (2023). Factors that influence the adoption of rehabilitation technologies: a multi-disciplinary qualitative exploration. J Neuroeng Rehabil.

[22] Selvanayakam S, Giovanoli S, Slot A (2025). I speak Tele outlines the design of a digitized dysarthria assessment. Sci Rep.

[23] Chandran S, Al-Sa’di A, Ahmad E Exploring user centered design in healthcare: a literature review.

[24] Good A, Omisade O (2019). Linking activity theory with user centred design: a human computer interaction framework for the design and evaluation of mHealth interventions. Stud Health Technol Inform.

[25] Rudzicz F, Namasivayam AK, Wolff T (2012). The TORGO database of acoustic and articulatory speech from speakers with dysarthria. Lang Resources & Evaluation.

[26] Etikan I, Musa SA, Alkassim RS (2016). Comparison of convenience sampling and purposive sampling. Am J Theor Appl Stat.

[27] Hustad KC (2007). Effects of speech stimuli and dysarthria severity on intelligibility scores and listener confidence ratings for speakers with cerebral palsy. Folia Phoniatr Logop.

[28] ggml-org/whisper.cpp. GitHub.

[29] Grier RA, Bangor A, Kortum P, Peres SC The system usability scale: beyond standard usability testing. Proceedings of the Human Factors and Ergonomics Society Annual Meeting.

[30] Konstantinidis S (2007). Computing the edit distance of a regular language. Inf Comput.

[31] Levenshtein VI (1966). Binary codes capable of correcting deletions, insertions, and reversals. Sov Phys Dokl.

[32] Xue W, van Hout R, Cucchiarini C, Strik H (2023). Assessing speech intelligibility of pathological speech in sentences and word lists: the contribution of phoneme-level measures. J Commun Disord.

[33] Kent RD, Weismer G, Kent JF, Rosenbek JC (1989). Toward phonetic intelligibility testing in dysarthria. J Speech Hear Disord.

[34] Weismer G, Jeng JY, Laures JS, Kent RD, Kent JF (2001). Acoustic and intelligibility characteristics of sentence production in neurogenic speech disorders. Folia Phoniatr Logop.

[35] Nylén F (2025). An acoustic model of speech dysprosody in patients with Parkinson’s disease. Front Hum Neurosci.

[36] Villain M, Cosin C, Glize B (2016). Affective prosody and depression after stroke: a pilot study. Stroke.

[37] Ross ED (2023). Affective prosody and its impact on the neurology of language, depression, memory and emotions. Brain Sci.

[38] Vaezipour A, Campbell J, Theodoros D, Russell T (2020). Mobile apps for speech-language therapy in adults with communication disorders: review of content and quality. JMIR mHealth uHealth.

[39] Wouda L, Boerma T, Gerrits E, Blom E (2024). First steps toward implementation of the online test battery LITMUS-NL: a usability and feasibility study. Perspect ASHA Spec Interest Groups.

[40] Kim Y, Thompson A, Lee SJ (2024). Does native language matter in perceptual ratings of dysarthria?. J Speech Lang Hear Res.

[41] Qian Z, Xiao K, Yu C (2023). A survey of technologies for automatic dysarthric speech recognition. EURASIP J Audio Speech Music Process.

[42] Qian Z, Xiao K (2023). A survey of automatic speech recognition for dysarthric speech. Electronics (Basel).

[43] Tröger J, Dörr F, Schwed L (2024). An automatic measure for speech intelligibility in dysarthrias-validation across multiple languages and neurological disorders. Front Digit Health.

[44] Bangor A, Kortum PT, Miller JT (2008). An empirical evaluation of the system usability scale. Int J Hum-Comput Interact.

[45] Attwell GA, Bennin KE, Tekinerdogan B (2022). A systematic review of online speech therapy systems for intervention in childhood speech communication disorders. Sensors (Basel).

[46] Green JR (2024). Artificial intelligence in communication sciences and disorders: introduction to the forum. J Speech Lang Hear Res.

[47] Shankar V, Ramkumar V, Kumar S (2022). Understanding the implementation of telepractice in speech and language services using a mixed-methods approach. Wellcome Open Res.

[48] Weidner K, Lowman J (2020). Telepractice for adult speech-language pathology services: a systematic review. Perspect ASHA Spec Interest Groups.

[49] Cetinkaya B, Twomey K, Bullard B, EL Kouaissi S, Conroy P (2024). Telerehabilitation of aphasia: a systematic review of the literature. Aphasiology.

