# Supplementary material for: Automatic Speech Recognition and Acoustic Analysis for Dysarthria Assessment in Telerehabilitation: User-Centered Design and Usability Study
Source: JMIR Form Res. 2026 Jul 3;10:e85230. doi: 10.2196/85230 (PMC13331071; doi:10.2196/85230)
Supplement: Multimedia Appendix 1 [file formative-v10-e85230-s001.docx]

## Supplementary Files

| ID | Benefit | FR | NFR | User Story |
| --- | --- | --- | --- | --- |
| N01 | Gives objective and quantifiable metrics | FR-01,  FR-02 | NFR-06 | Provide me with core acoustic parameters so I can quantify voice quality. |
| N02 | Removes therapist bias and gives measurable progress data | FR-01 | NFR-01 | As an SLP I want an automatic intelligibility score so I can validate my judgment. |
| N03 | Therapists save time (Praat deemed too time consuming) | FR-02 | NFR-02 | SLPs have no time to learn Praat and extract relevant feature from speech analysis softwares. |
| N04 | Lets therapists work on results when they have time | FR-02,  FR-01 | NFR-02,  NFR-04 | As an SLP I want the analysis right after the call so I can plan the next session. |
| N05 | Quick interpretation | FR-04 | NFR-04 | Show me a colour code so I instantly know if a value is within normal range. |
| N06 | Ensures reliable sessions | FR-09 | NFR-03 | I need the system to normalise loudness for the recording. |
| N07 | Shareable evidence for patients & therapists | FR-06a,  FR-06b | NFR-01,  NFR-04,  NFR-12 | I need a report about the patient to send to the patient or the therapist. |
| N08 | Tracks rehabilitation trajectory over sessions | FR-07 | NFR-01,  NFR-05,  NFR-12 | As an SLP I want a graph across sessions so I can show progress to the patient. |
| N09 | Asynchronous assessments when possible | FR-11 | NFR-02,  NFR-07 | For assessments I prefer to upload and review later. |
| N10 | Enables detailed postsession listening | FR-10 | NFR-02,  NFR-04 | I like to listen back to the recording to double-check articulation errors. |
| N11 | Prevents unusable recordings (tips if ”shh/fff”are cut) | FR-08 |  | Warn me if the platform noise suppression is ruining high-frequency sounds. |
| N12 | Enables on-the-fly session adjustments | FR-03 | NFR-03 | While I’m online with a patient I’d like to see acoustic parameters to adjust the therapy on the fly. |
| N13 | Minimises variability between sessions | FR-09 |  | Calibrate the mic before each sessions to account for intensity thresholds. |
| N14 | Synchronous error counting during therapy | FR-18 | NFR-03,  NFR-02 | I want live error percentages during articulation tasks. |
| N15 | Dysphonia-specific acoustic markers | FR-12 | NFR-06 | NFR-06 Give me jitter, shimmer, voice-breaks to characterize dysphonia. |
| N16 | Detect strong initial glottal attack (glottisslag”) | FR-12 | NFR-06 | Flag abnormally loud first onset in sustained phonation or speech. |
| N17 | Fluency/disfluency metrics for aphasia | FR-14 | NFR-06 | Show rate, pauses, blocks, repetitions to quantify fluency. |
| N18 | Control over which assessment to analyze, automated within it | FR-13 | NFR-07 | Let me pick the assessment and run the matching analysis automatically. |
| N19 | Paragraph read-aloud: word-level comparison and percentage correct | FR-01 | NFR-06 | Insert a paragraph to read and compare which words were mispronounced. |
| N20 | Arabic articulation support (incl. Amaria Test) | FR-15,  FR-16 | NFR-10,  NFR-11 | Support Arabic phonemes and my standardized Arabic articulation test. |
| N21 | First-session report | FR-06a,  FR-06b | NFR-01,  NFR-11 | After the first session I need a baseline report |
| N22 | Interdisciplinary updates every 3–6 months | FR-07 | NFR-11 | Compile a periodic summary for the wider care team. |
| N23 | Connectivity doesn’t stop therapy | FR-17 | NFR-08 | If the connection drops, keep recording locally and sync later. |

Table 1: SLPs needs and requirements

| ID | Title | Brief Description |
| --- | --- | --- |
| FR-01 | Auto intelligibility score | Automatically compute intelligibility for each recording |
| FR-02 | Auto acoustic analysis | Automatically extract core acoustic features from voice recording |
| FR-03 | Live metrics during session | Present each key metric with a green / yellow / red status to allow instant interpretation against normative ranges. |
| FR-04 | Color-coded norms | Color/status coding to show whether values fall within normal ranges. |
| FR-06a | Shareable report | Generate a clinician report with export (PDF/CSV). |
| FR-06b | Shareable report | Generate a patient-friendly report with export (PDF/ CSV). |
| FR-07 | Longitudinal tracking | Graph metrics across sessions, highlight trends and progress. |
| FR-08 | Recording QA & tips | Detect high-frequency cut/AGC/noise suppression (e.g.,”shh/fff”), surface actionable tips. |
| FR-09 | Loudness normalization | Normalize voice recording for session for consistency |
| FR-10 | Post-session reviewer | Player with waveform/spectrogram + time-stamped notes for detailed relistening. |
| FR-11 | Asynchronous assessments | Upload assessments for asynchronous processing (therapist reviews later). |
| FR-12 | Dysphonia markers | Quantify jitter, shimmer and voice-break counts |
| FR-13 | Protocol-aware analysis | Therapist selects assessment, system runs the matching analysis automatically. |
| FR-14 | Fluency/disfluency (aphasia) | Speech rate (WPM), pause segmentation, block/stop detection, repetitions/fillers. |
| FR-15 | Multilingual articulation | Phoneme/IPA support including Arabic |
| FR-16 | Custom test builder | Define/edit standardized tests and scoring rubrics (e.g.,”Amaria Test”). |
| FR-17 | Connectivity-resilient capture | Local-first recording, auto-sync on reconnection, connectivity status indicator. |
| FR-18 | Live error % (articulation) | Real-time error counting/percentages during synchronous articulation tasks. |

Table 2: Functional Requirements

| ID | Title | Brief Description |
| --- | --- | --- |
| NFR-01 | Clinical consistency - objectivity | Standardized scoring, results are consistent across therapists. |
| NFR-02 | Speed - low friction | Minimal clicks, fast analysis turnaround after upload. |
| NFR-03 | Real-time - calibration robustness | Low-latency live metrics; device-agnostic levels despite AGC; stable performance. |
| NFR-04 | Interpretable – accessible UI | Clear legends, colorblind-safe palettes, thresholds tied to norms. |
| NFR-06 | Metric validity | Accuracy/benchmarking vs accepted tools/ground truth for the targeted metrics. |
| NFR-07 | Ease of adoption | Onboarding/help in-product, little to no prior software training required. |
| NFR-08 | Network resilience | Network down handling, no data loss |
| NFR-09 | Privacy - compliance | GDPR/HIPAA-ready, encryption, consent, role-based access, retention policies. |
| NFR-10 | Internationalization | Full i18n inclusion |
| NFR-11 | Interoperability & export | PDF/CSV exports. |
| NFR-12 | Auditability | Timestamped analyses |

Table 3: Non-Functional Requirements

| ID | SLP Feedback | Refined | Participant ID |
| --- | --- | --- | --- |
| I01-001 | Patient 2: In the sentence section some words were missing in the transcription although they were clearly pronounced in the recording - which amounted to all the ”not understandable” words and to all the wrong phonemes, so here the analysis didn’t seem to work. | Fix alignment transcription reference | SLP003 |
| I01-002 | Not sure why, but the first set of files sent to me did not upload | Fix upload file | SLP009 |
| I01-003 | Not sure what the issue was, but I was using files sent to me rather than from my desktop files | Fix upload file | SLP009 |
| I01-004 | Use of the term ”misspelled” words is confusing. So intelligibility should not be tied to how the SLP types in the word. Calling the errors ”misspelled” makes me think that the SLP made the errors. | Change term ”misspelled” | SLP009 |
| I01-005 | I like the ability to select a segment to transcribe. But one should be able to listen to the segment and add the words at the same time. I have to transcribe on a separate paper, then type them in. | Listen to the segment while inserting reference | SLP009 |
| I01-006 | Allow editing of transcribed sentences/words after adding them without having to delete and reselect the recording. | Allow editing of transcribed sentences/words after adding them without having to delete and reselect the recording. | SLP004 |
| I01-007 | When I uploaded the clip the time measurement should be shown directly under it (min and sec), so I don’t have to guess where the word section ends and the sentences begin, but I can choose directly based on the recording time. | Display time range (min/sec) | SLP003 |
| I01-008 | And to be honest, we don’t know the IPA Chart by heart, it would be really great to have an IPA chart in an info box to look the different sounds up (especially if we are talking about using this application as a non-native speaker of English). | Add an IPA table in an info box as a helper | SLP003 |
| I01-009 | Also the voice variability could be added (as in intonation, which is also often affected, patients tend to speak very monotone). | Voice base frequency variability | SLP003 |
| I01-010 | Patient 1: word section: The bad rating of the sound /r/ wasn’t right, the sound wasn’t mispronounced that much, maybe the different kinds of r-sounds in English might play a role here. The vowels were more mispronounced than the score showed. Also the transcription of ”hair” as ”hat” I don’t get, I cannot really hear a ”t” at the end. | Fix /r/ phonemes transcription | SLP003 |
| I01-011 | Show the position of the mispronounced sound in the word (initial-medial-final) | Show the position of the mispronounced sound in the word (initial-medial-final) | SLP004 |
| I01-012 | In the sentence part the intelligibility was 0% – but there were some intelligible words there (like ”a cold shower”), so it was really bad but not 100% nonintelligible – that is a bit misleading. | Take into account correct words in the sentences in the calculation of intelligibility score, display if the word is correct, substitute or delete | SLP003 |
| I01-013 | When I selected the clip to make it longer, to include all sentences, it suddenly shifted right. Could be corrected easily, but happened every time when I first selected the clip length. | Fix shifting region | SLP003 |
| I01-014 | There should also be a way to slow down the recording. I also like to listen to something slower to help me process the sounds/phonemes. | Play recording at different speeds | SLP009 |
| I01-015 | Show if the phoneme is isolated or clustered (e.g., “stew” vs “sew”). | Display phoneme whether it is isolated or clustered (e.g., “stew” vs “sew”). | SLP009 |
| I01-016 | It would be very interesting to also have an analysis of the consonant clusters, because some sounds were pronounced wrong in clusters but not outside of clusters. | Add analysis on the consonant-clusters level | SLP003 |
| I01-017 | It would also be helpful if the system could draw conclusions about voice quality (hoarseness, roughness, etc.) as well as tempo and volume. | Include voice quality (hoarseness, roughness, etc.), tempo and volume | SLP010 |
| I01-018 | With patient 1 you could hear that there were problems with the pauses between words (as in there were none, the words often melted together which makes it more unintelligible). This is often a problem in dysarthria: too long or too short breaks between words, which melts words or makes it hard to get the content of sentences. I wonder if that could be analyzed as well? | Measure and analyze the duration of breaks between words | SLP003 |
| I01-019 | I typed in “write” which should have counted as correct for “right”. We don’t consider spelling, but phonemic production of “wr” is the same as “r”. And same for “dagger” – I typed “dager” which could be the same production but spelled wrong. Another example: if I typed “alfa”, would that be an error as the “correct” spelling is “alpha”? Or I heard the word “fuhval” – how do I type a schwa sound? Need to be able to indicate distorted vowels; I would probably transcribe using diacritics and phonetic transcription. | Identify phonetic production and count similar phonetic as good for intelligibility calculation | SLP009 |
| I01-020 | Is there the ability to save each recording to go back to? I wanted to hear the productions again after analysis, but had to upload it again. | Ability to save each recording and playback after analysis | SLP009 |
| I01-021 | System should use phonetic transcription to be able to indicate distortions or alternative productions. I would need to see an updated version of the program before recommending to a colleague. | Display phonemes distortions and alternative productions | SLP009 |
| I01-022 | Would like to use it for research subjects as well. Potential adaptation to Italian in the future. | Italian adaptation | SLP002 |
| I01-023 | The transcription could be improved a little. The system did not capture all the words of the severely affected patient as they were mumbled. But when I listen to the recordings, I can hear the words. | Improve transcription model | SLP010 |

Table 4: SLP feedback cycle 1

Figure 1. I-speak style guide
